# A new narrative for social immunity research in termites

**DOI:** 10.1016/j.treopn.2026.08.007

**Published:** 2026-10

**Authors:** Aryel C. Goes, Erin L. Cole, Ives Haifig, Dino P. McMahon, Michael Poulsen

**Affiliations:** 1 Department of Evolution, Ecology and Organismal Biology, Museum of Biological Diversity, The Ohio State University, Columbus, OH 43212, USA; 2 Section for Ecology and Evolution, Department of Biology, University of Copenhagen, 2100 Copenhagen East, Denmark; 3 Centro de Ciências Naturais E Humanas, Universidade Federal Do ABC – UFABC, São Bernardo do Campo, SP 09606-070, Brazil; 4 Institute of Biology, Freie Universität Berlin, 14195 Berlin, Germany; 5 Department for Materials and Environment, BAM Federal Institute for Materials Research and Testing, 12205 Berlin, Germany

**Keywords:** altruism, hygiene, Isoptera, inclusive fitness, social organisation

## Abstract

Closely related workers of insect societies gain indirect fitness benefits by performing costly colony-defence tasks, resulting in the evolution of hygienic traits. In ants and in many bees and wasps, strict division of reproductive labour implies clear predictions for the expression of hygienic traits. Termites, however, differ from such canonical hymenopteran social patterns because they exhibit a high degree of developmental plasticity, with worker castes varying in reproductive potential. Termites therefore represent an ideal study system to explore the evolutionary origins of altruistic hygienic traits. In this review, we identify robust categories of social organisation and summarise our current understanding of hygienic trait expression in light of fitness prospects in termites. We also outline key gaps and hypotheses for future work, using a conceptual framework built around developmental flexibility and fitness to enable new insights into the evolution and expression of social immunity.

## Significance

Current social immunity theory does not integrate how fitness prospects affect individual expression of altruistic traits. Using termites, we develop an ontogenetic framework for hypothesis-driven testing of the expression and evolution of social immune traits in relation to indirect and direct fitness potential. Mapping current knowledge onto this framework illustrates substantial research gaps, the need for work on ecologically relevant pathogens, and opportunities for significant comparative analyses of hygienic traits across the Isoptera.

## Social organisation and immunity

Some of the most dramatic organisational changes during evolution involved shifts from individual to cooperative lifeforms. Referred to as **major evolutionary transitions** (see Glossary), these organisational shifts enabled **reproductive altruism**, where fitness gains from helping kin (indirect fitness) are promoted over fitness gained from reproducing (direct fitness) 1, 2, 3, 4. In extreme cases, this gave rise to **superorganismal** insect societies with permanently segregated reproductive and sterile castes, such as ants and honeybees, where inclusive fitness interests and colony success are fully aligned 4, 5. However, in societies with more flexible forms of reproductive division of labour, such as termites, the expression of altruism may be more context-dependent [4]. Frameworks linking these traits are lacking, despite being crucial for understanding the conditions that lead to the evolution of altruistic traits.

Immunity can be viewed as an important mechanistic link between reproductive flexibility and altruism [6]. Both conflict mediation and pathogen defence are essential for maintaining individual fitness interests while preserving group integrity 7, 8. In this context, immune systems evolved not only to defend against exogenous threats but also to limit selfish exploitation 9, 10. This role extends to transitions in individuality, such as the emergence of multicellularity and the origins of sociality, where the latter is marked by colony-level protection that contributes to group cohesion (i.e., **social immunity** [11]). From an **inclusive fitness** perspective (Box 1), the evolution and expression of altruistic social immune traits are expected to depend on the alignment of fitness interests among group members [15]. In lineages where relatedness is high and inclusive fitness interests are fully aligned, we expect altruistic social immune traits to be unconditional, and individuals to express costly behaviours to help protect the colony. In contrast, in systems where reproductive cooperation is facultative (e.g., cooperative breeders), social immunity may be conditional. In such cases, defensive traits are expected to be expressed when the benefits of helping kin outweigh any direct fitness costs to the individual [15].

Box 1 **Inclusive fitness theory**

**Inclusive fitness theory** is grounded in Hamilton’s rule, which predicts that individuals should be selected to help other individuals in proportion to their shared relatedness, as long as fitness costs do not exceed the fitness benefits of helping (i.e., when helping maximises their total fitness 1, 2, 3, 12, 13, 14). This simple rule can be applied to explain sociality and altruism, as illustrated in the scenarios given below. It is, therefore, not necessary to invoke relatedness asymmetries as seen in social Hymenoptera 1, 2, 3 or the subjective superiority of human-like societies. This prediction can be illustrated in three scenarios:

i) In a monogamous mating system with chromosomal sex determination, individuals share 50% of their genes with offspring and 50% of their genes with their siblings. In this scenario, ecological and physiological constraints will dictate whether an individual gains greater evolutionary fitness through the production of offspring or through assisting their parents in raising more siblings. For instance, producing one offspring yields less fitness than helping produce two siblings, assuming monogamy is strict and siblings share at least 50% of their genes.
ii) In inbred systems, where individuals mate with parents or siblings, the percentage of shared genes in the resulting offspring can exceed 50%. Unless inbreeding depression or ecological factors impose negative selection, such a system should favour altruism that results in the production of more siblings over pursuing direct fitness, since offspring will only share 50% of their genes, whereas siblings would share more than 50%.
iii) In systems without strict monogamy or where unrelated individuals breed communally, siblings or group members share, on average, less than 50% of their genes. In this case, individuals gain greater fitness from producing offspring than from helping siblings reproduce.

In all three scenarios, altruism is predicted by both the percentage of shared genes (inclusive fitness) and the ecological and physiological conditions that dictate the costs of altruism. Importantly, natural selection acts through the cumulative outcomes of successive generations. Hence, for altruism (or any phenotypic trait) to occur and become fixed in a population, the altruistic behaviour must first evolve or arise through mutation, the contextual conditions that favour altruism must be present, and these conditions must be maintained over successive generations.

**Evolutionary fitness:** The total number of genes an individual spreads in the population.

**Direct fitness** (*D*) is the propagation of a gene within an individual’s offspring.

$$D=r_{\text{offspring}\times \text{total offspring}}$$

**Indirect fitness** (*I*) is fitness obtained through the propagation of shared genes present in the offspring of relatives, such as parents or siblings.

$$I=r_{\text{relative}\times \text{total relatives}}$$

Inclusive Fitness=D+I

**Hamilton’s rule**

rB>C

*r* is the **relatedness coefficient**, or the percentage of shared genes. The relatedness coefficient between parents and offspring is usually *r* = 0.5 because they typically share 50% of their chromosomal genes. *r* between a parent and second-generation offspring would be 0.25.

*B* denotes **benefits** in terms of evolutionary fitness, which occur when an action increases an individual’s reproductive output and/or the reproductive output of relatives.

*C* denotes **costs** in terms of evolutionary fitness, which occur when an action reduces direct reproductive output.

Alt-text: Box 1

Unlike ants and honeybees, in which unconditional investment in social immunity may be facilitated by a stricter segregation between worker and reproductive castes, termites exhibit wide variation in caste development and reproductive potential. As **hemimetabolous** insects, termites exhibit **paedomorphic** developmental traits, with individuals retaining juvenile characteristics until they reach the reproductive stage [16]. Thus, variation in social organisation and developmental complexity across castes, species, and lineages affects individual fitness potential and could therefore impact the expression of altruistic traits 12, 16, 17, 18. This makes termites an ideal study system for testing how direct and indirect fitness opportunities shape investments in social immunity. While recent studies have addressed some expectations for conditional social immunity 15, 19, it is still unclear how ontogeny, reproductive potency, and social organisation affect the expression of such defensive traits.

Here, we use social immunity in termites to develop a conceptual framework for trait expression predictions based on ontogenetic potency. Our goal is to clarify this relationship by identifying robust categories of termite social organisation that allow for inferences about the potential for indirect and direct fitness benefits and costs linked to social immunity expression at the individual level. We then explore whether conditional versus unconditional fitness potential can be used to derive testable predictions of hygienic expression. Finally, we synthesise current knowledge and gaps in termite social immunity research and outline hypothesis-driven priorities for future work on the relationship between social organisation and immunity.

## Social organisation and social immunity predictions based on fitness potential

Many hygienic mechanisms in social insects, such as self- and allogrooming, have been reported for solitary and subsocial species [20], suggesting that important components of social immunity evolved before the transition to advanced sociality. Group living favours such defences as they decrease exposure to socially transmitted pathogens. Within the Blattodea, self-grooming occurs in cockroaches [21] that exhibit diverse forms of aggregation, kin recognition, and collective behaviours [22]. Thus, several mechanisms later incorporated into the social immune systems of termite lineages were likely present in solitary, communal, or family-living cockroach ancestors [20] to mitigate infection costs emerging from social contact.

The apterous (wingless) **subsocial** woodroaches of the genus *Cryptocercus* are particularly informative for the evolution of hygienic strategies because they represent the extant sister lineage to termites within the Blattodea [23]. They also retain the ancestral hemimetabolous developmental pathway of linear progressive moults before reaching adulthood 24, 25. *Cryptocercus* lives in biparental family groups where dependent offspring receive resources from their parents [26]. Group living increases physical interactions for which hygienic traits should allow direct and indirect fitness benefits through improved protection of individuals and kin. Immune resource allocations should be maximised to favour individual survival and reproduction, including when conditional expression of social immunity is favoured [15]. Consequently, parents are expected to protect offspring collectively to maximise resource efficiency. Offspring, by contrast, retain future reproductive potential and should thus express costly hygienic behaviours more selectively. Such behaviours may be favoured when (1) they reduce the offspring’s own risk of infection, (2) they protect closely related siblings or parents, or (3) they improve both direct and indirect fitness benefits. In *Cryptocercus*, immune repertoires include allogrooming of siblings [27] and Gram-negative binding proteins (GNBPs) to protect against infections [28]; this is consistent with the hypothesis that components of social immune traits were present in the ancestor of the termites and potentially central to the evolution of sociality.

In obligately social species, indirect fitness benefits become more important, and direct fitness components will depend on the potential for individual reproduction. In many termite species, individuals retain developmental plasticity and may become reproductives under certain conditions. Based on past studies on caste differentiation, social organisation, and reproduction in termites (Table S1 in the Supplemental information online), we identify three distinct categories of social organisation with concomitant differences in predicted fitness potential (Figure 1A–C). We use this framework to examine variation in social immunity strategies, focusing on the extent to which colony members are predicted to express social immunity: conditionally, unconditionally, or not at all.

### Linear and totipotent

Linear termite social organisation is based on an ontogenetic scheme that is thought to represent the ancestral condition in termites 32, 33 and it is most similar to standard hemimetabolous development. However, it has several key deviations: (i) flexible regressive, stationary, or progressive moults; (ii) larval and nymphal stages that can develop into neotenic reproductives that supplement or replace the original colony founders [29]; (iii) a sterile soldier caste [34]; and (iv) mature colonies of some species can produce intercaste soldiers with enlarged mandibles and functional reproductive organs 35, 36, 37, 38 (Figure 1A).

**Figure 1:**
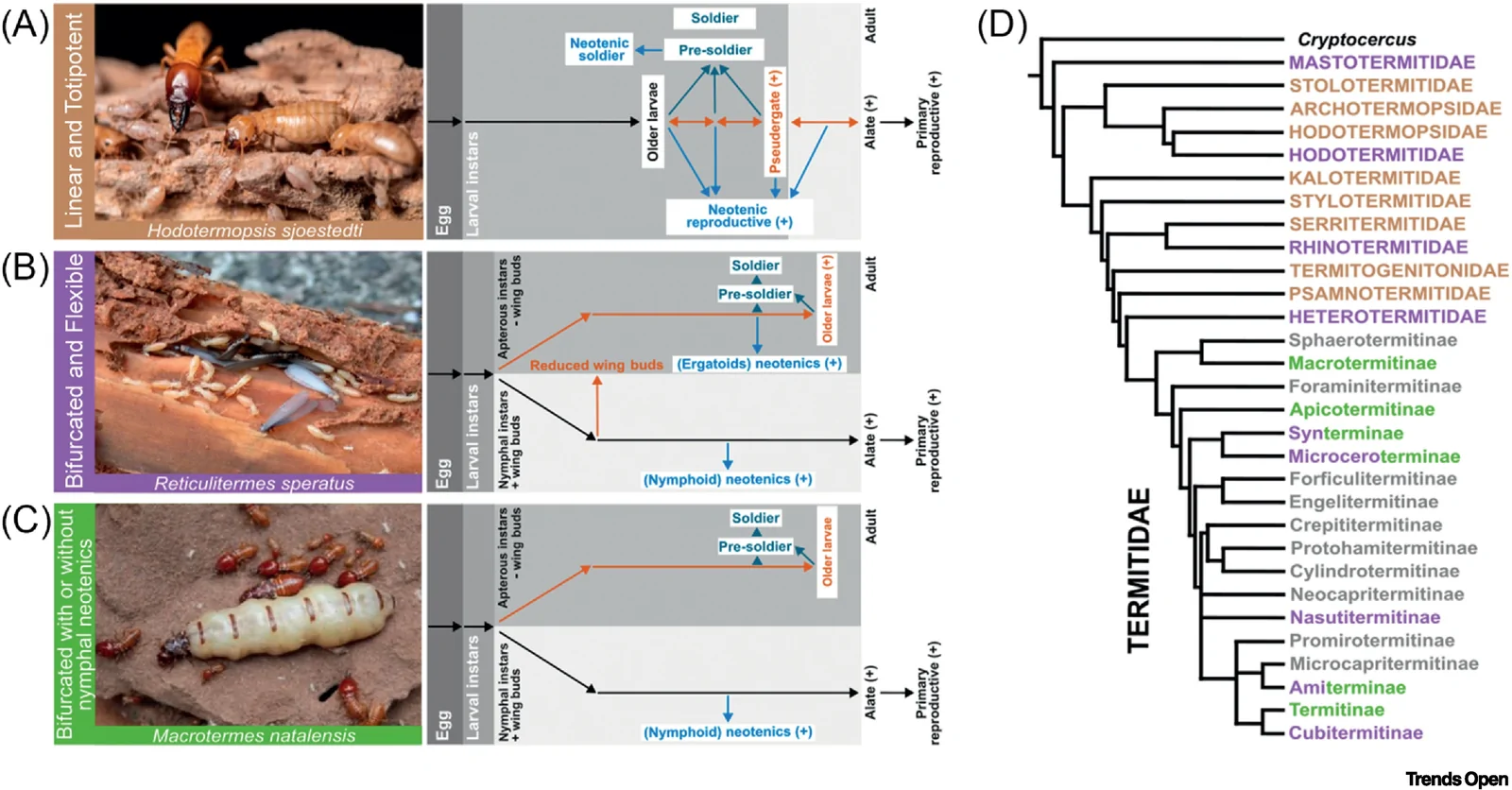
Social organisation and direct fitness potential of termite castes. Panels A–C show representative images of three termite species with distinct social structures, their developmental pathways, and the fitness prospects that colony members experience. (A) Linear and totipotent, which is ancestral at its core but also includes developmental flexibility via progressive, regressive, and stationary moults, as well as the presence of pre-imaginal reproductives (neotenics) and soldiers. Image credits: Aleš Buček, iNaturalist Photo 101759370, all rights reserved. (B) Bifurcated and flexible. Individuals diverge early and irreversibly in their development into lines with either immature wing buds (nymphal line) or no wing buds (worker line). Neotenic reproductives may also be derived from the worker line (i.e., ergatoid neotenics [29]), although their occurrence in natural colonies is typically very low (where investigated) and therefore workers of such species are effectively (functionally) sterile. Note that the definition of ergatoids in termites differs from that used in the Hymenopteran literature, where ergatoids strictly come from the queen developmental pathway and are analogous to termite nymphoid and adultoid neotenics [30]. Image from https://commons.wikimedia.org/wiki/File:Reticulitermes_speratus_Colony01.jpg. (C) Bifurcated with or without nymphoid neotenics. Here, the worker-line is cut off from direct reproduction and consequently locked into unconditional altruism, irrespective of whether reproductives can produce nymphal neotenics or not. Image credits: https://www.smithsonianmag.com/smithsonian-institution/could-ants-termites-and-fishes-carry-lessons-to-make-humans-better-farmers-180980085. (D) The three categories outlined in A–C are colour-coded on a schematic termite phylogeny based on Hellemans *et al*. [31]. The phylogeny includes the closest extant relative of termites (*Cryptocercus*), 13 termite families (bold), and 18 termite subfamilies within the Termitidae (not bold). Subfamilies in grey do not have data available to allow their assignment to a category (literature in support of categorisations can be found in Table S1). The categories assigned for the families and subfamilies are tentative, as not all of the species diversity and caste systems are known within these groups. Panels A–C show representative images of three termite species with distinct social structures, their developmental pathways, and the fitness prospects that colony members experience. (A) Linear and totipotent, which is ancestral at its core but also includes developmental flexibility via progressive, regressive, and stationary moults, as well as the presence of pre-imaginal reproductives (neotenics) and soldiers. Image credits: Aleš Buček, iNaturalist Photo 101759370, all rights reserved. (B) Bifurcated and flexible. Individuals diverge early and irreversibly in their development into lines with either immature wing buds (nymphal line) or no wing buds (worker line). Neotenic reproductives may also be derived from the worker line (i.e., ergatoid neotenics [29]), although their occurrence in natural colonies is typically very low (where investigated) and therefore workers of such species are effectively (functionally) sterile. Note that the definition of ergatoids in termites differs from that used in the Hymenopteran literature, where ergatoids strictly come from the queen developmental pathway and are analogous to termite nymphoid and adultoid neotenics [30]. Image from https://commons.wikimedia.org/wiki/File:Reticulitermes_speratus_Colony01.jpg. (C) Bifurcated with or without nymphoid neotenics. Here, the worker-line is cut off from direct reproduction and consequently locked into unconditional altruism, irrespective of whether reproductives can produce nymphal neotenics or not. Image credits: https://www.smithsonianmag.com/smithsonian-institution/could-ants-termites-and-fishes-carry-lessons-to-make-humans-better-farmers-180980085. (D) The three categories outlined in A–C are colour-coded on a schematic termite phylogeny based on Hellemans *et al*. [31]. The phylogeny includes the closest extant relative of termites (*Cryptocercus*), 13 termite families (bold), and 18 termite subfamilies within the Termitidae (not bold). Subfamilies in grey do not have data available to allow their assignment to a category (literature in support of categorisations can be found in Table S1). The categories assigned for the families and subfamilies are tentative, as not all of the species diversity and caste systems are known within these groups.

We predict that strategies optimising fitness should differ by caste, colony ontogeny, and ecological factors. During the incipient colony stage, all offspring produced by the primary royal pair are inherently limited to inclusive fitness benefits, regardless of caste or physiological capacity. Accordingly, we predict rudimentary social immunity as a form of parental care and/or **transgenerational immune priming**, initially expressed by the founding pair and later by nestmates. As colonies grow, we expect a mixture of strategies that minimise colony-level risks while maximising sibling production from **primary reproductives** and **neotenics**. Precise strategies may differ by caste, as sterile soldiers are predicted to always invest in colony defences and social immunity, while reproductives and workers should balance direct and indirect reproduction with altruistic hygiene. Ecological conditions, particularly for species that inhabit their food source, such as drywood and dampwood termites, should modulate strategies depending on resource availability and pathogen pressures.

### Bifurcated and flexible

Bifurcated development splits individuals after the initial larval instars into those with wing buds (+wing buds; the nymphal line) and those without wing buds (−wing buds; the worker line) (Figure 1B). The nymphal line follows an ancestral hemimetabolous trajectory, with progressive moults leading to reproductive maturity. The worker line retains some developmental flexibility, including the potential to become an **ergatoid neotenic** with the capacity for direct fitness [39] (Figure 1B). However, ergatoid production is rare and conditional 17, 18, 40, meaning that most workers are constrained to lifelong sterility despite residual totipotency 17, 41. Selection on worker-line individuals is therefore expected to primarily reflect indirect fitness returns. Hence, the expression of costly social immune traits is traded-off with conditional colony-level prospects for workers becoming ergatoids. We predict that investment in costly social immune traits will correlate negatively with the frequency of ergatoids, with costly social immune traits emerging in species, or under ecological conditions, where workers are more reliably sterile and opportunities for ergatoid development are rare. Notably, estimates of ergatoid occurrence are lacking due to limited research effort and difficulties in detection, which should be a priority for future work, including in connection with predictions for social immune expression.

### Bifurcated with or without nymphal neotenics

Development is also bifurcated, with workers and soldiers developing from the –wing bud line, whereas **alates,** and in some lineages **nymphoid** **neotenics,** arise from the +wing bud line [42] (Figure 1C). A variant of this type of development is superorganismality (*sensu* [12]), which lacks neotenics and has alates produced from the +wing bud line (Figure 1C). Individuals on the path to becoming alates may not be expected to express altruistic traits. For nymphoid neotenics, prospects for direct reproduction are ambiguous, and they may conditionally express social immunity depending on the likelihood of realising direct fitness [15]. In this category, the worker line has no potential for direct fitness and should display unconditional social immunity. The fitness prospects of individuals in this category thus align with the term superorganismality, even if its applicability remains debated in the termite literature [18].

### Social organisation systems across the termite phylogeny

Mapping the categorical framework onto the most recent termite phylogeny reveals that we have yet to obtain a robust understanding of social organisation in many extant termite lineages [31] (Figure 1D). Most importantly, these categories are not monophyletic, implying that they are not the product of sequential, progressive changes in social complexity over evolutionary time 33, 43. Multiple lineages have independently evolved comparable forms of social organisation, each representing examples of increased polyphenism via the emergence of a specialised worker caste that is functionally, though not obligately, sterile (*sensu* [18]). The implications of this are the following: (i) variation in social immune trait expression is expected, (ii) there is a need to fill gaps in our understanding of termite social organisation, (iii) future work may reveal new categories or variation within the currently recognised categories, and (iv) there are ample opportunities for comparative analyses of convergently evolved traits. Notably, although social organisation remains poorly resolved for several termite lineages, this does not compromise our principal predictions, even if it limits the extent to which rigorous comparisons can currently be made.

## Does the distribution of social immune traits in termites fit social organisation?

A synthesis of the current termite literature on social immunity (Text S1 and Table S2 in the Supplemental information online) reveals that traits are sporadically and unevenly detected and subject to severe taxonomic bias (Figure 2A). Eleven families or subfamilies have not been studied, and the family Termitidae is particularly poorly explored. Only a few (sub)families have had more than five traits examined (Heterotermitidae, Macrotermitinae, Kalotermitidae, and Nasutitermitinae); several have reports on no traits or only a single trait (e.g., Termitinae and Cubitermitinae); and only a handful of traits (i.e., grooming, genes involved in defences, antimicrobial secretions, and GNBPs) have been examined in more than a few studies (e.g., in Hodotermitidae, Mastotermitidae, and Archotermopsidae). Likely explanations for this include preferential reporting of confirmatory findings and a bias towards work on termite species that are most relevant to humans. In line with this assertion, economically important pests in the Heterotermitidae and Kalotermitidae have been the subject of extensive experimental work on, for example, resistance and resilience to pathogens 45, 46. Lastly, conspicuous, abundant, and ecologically prominent taxa (e.g., Macrotermitinae and Nasutitermitinae) have received more attention than inconspicuous soil-dwelling taxa (e.g., Termitinae, Cubitermitinae, and Hodotermitidae).

Behavioural, chemical, and immune gene defences are among the most frequently assessed traits. Most of these are likely ancestral, as they appear universal (e.g., grooming, GNBPs, and cellular immune responses), while others may be restricted to one or a few termite lineages (e.g., antimicrobial materials and immune roles of soldiers) (Figure 2A). Nevertheless, even readily observable behaviours may be overlooked when not explicitly explored. For instance, corpse management has received little attention despite cannibalism, corpse retrieval, burial, and isolation being described as critical components of colony defence 47, 48, 49. Their expression may vary with feeding and nesting ecology, since cannibalism can facilitate nutrient recycling while nest architecture and access to soil or building materials can affect opportunities for burial or self-isolation 48, 49, 50, 51. Other subtle or context-dependent traits, such as aggression, social immunisation, and avoidance, are similarly overlooked (Figure 2A). Consequently, uneven representation of traits across termite taxa likely reflect both ecological variation and research biases.

**Figure 2:**
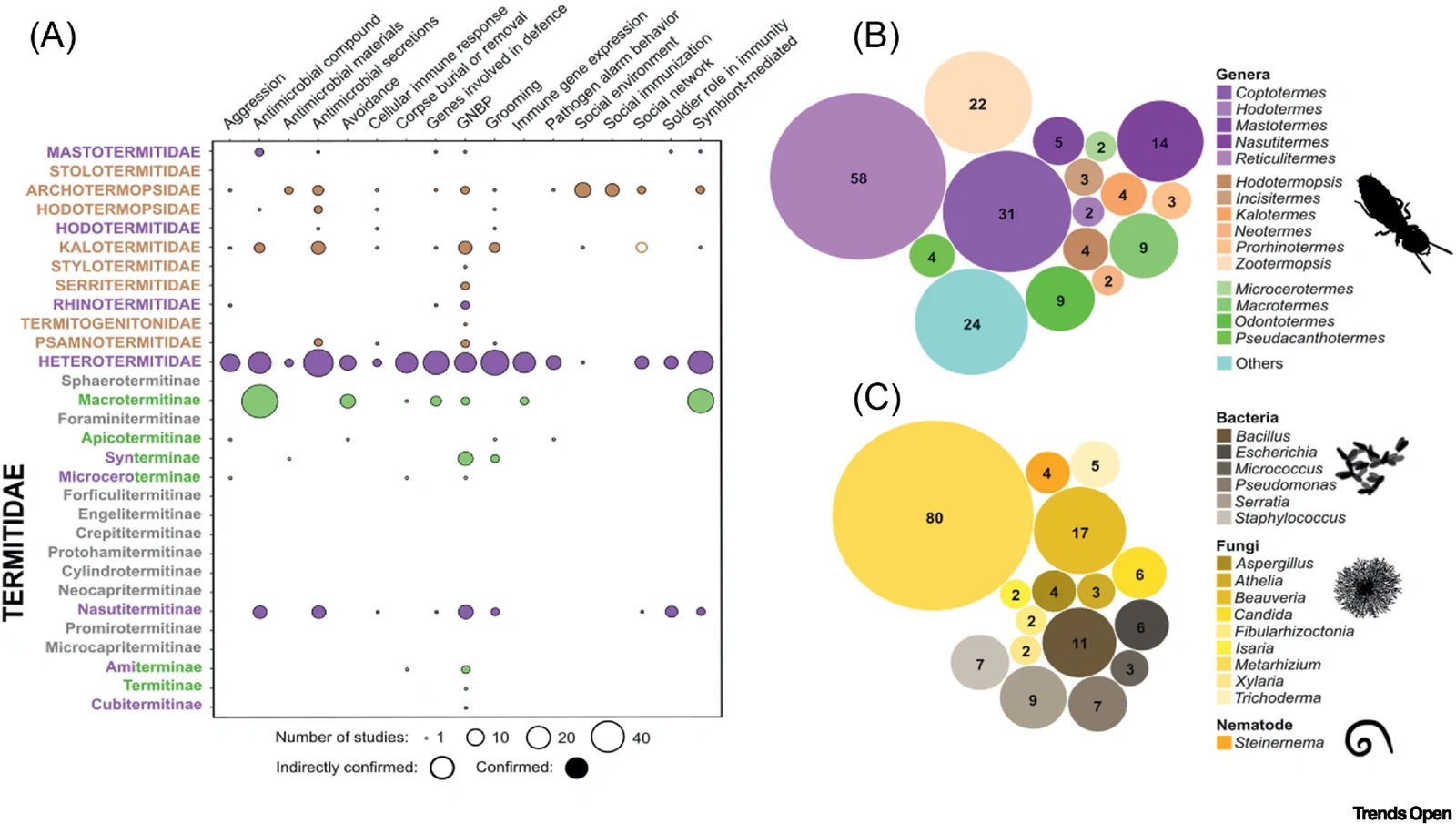
Social immunity traits and study effort across termite lineages. (A) Summary of social immune traits for which there is evidence (solid circles) or indirect confirmation (hollow circles). Circle sizes indicate the number of studies confirming each trait for a given (sub)family, and colour denotes the social organisation strategy of the respective taxon (brown: linear and totipotent; purple: bifurcated and flexible; green: bifurcated with or without nymphal neotenics; purple and green: both categories are represented; refer to Figure 1). If independent experiments on the same termite host provided contrasting results, evidence in favour of the trait being present is indicated; Table S2 provides detailed outcomes. (B) The distribution of experiments by focal termite genus is represented as a bubble chart. Bubble size corresponds to study count, and colour shades reflect social organisation (Figure 1). (C) Pathogen taxa used in social immunity experiments are shown. Bubble size represents the number of studies per pathogen genus, and colour shades indicate pathogen group (yellow shades: fungi; brown shades: bacteria; orange: nematodes). The literature search method and traits investigated are explained in Text S1, and references are given in Table S2. The figure was made in R Studio v. 3.6.0 [44] and formatted with Inkscape v. 1.4.3. GNBP: Gram-negative binding protein. (A) Summary of social immune traits for which there is evidence (solid circles) or indirect confirmation (hollow circles). Circle sizes indicate the number of studies confirming each trait for a given (sub)family, and colour denotes the social organisation strategy of the respective taxon (brown: linear and totipotent; purple: bifurcated and flexible; green: bifurcated with or without nymphal neotenics; purple and green: both categories are represented; refer to Figure 1). If independent experiments on the same termite host provided contrasting results, evidence in favour of the trait being present is indicated; Table S2 provides detailed outcomes. (B) The distribution of experiments by focal termite genus is represented as a bubble chart. Bubble size corresponds to study count, and colour shades reflect social organisation (Figure 1). (C) Pathogen taxa used in social immunity experiments are shown. Bubble size represents the number of studies per pathogen genus, and colour shades indicate pathogen group (yellow shades: fungi; brown shades: bacteria; orange: nematodes). The literature search method and traits investigated are explained in Text S1, and references are given in Table S2. The figure was made in R Studio v. 3.6.0 [44] and formatted with Inkscape v. 1.4.3. GNBP: Gram-negative binding protein.

Studies typically also focus on sets of traits that are assumed to be representative of social immunity in insects 11, 52. Specifically, grooming (i.e., allogrooming) is a prominent strategy in termites because of its prophylactic role in reducing pathogen load, mitigating infectious disease exposure and transmission, and reducing the need for physiological investments in individual immune responses 53, 54, 55, particularly when individuals face weak innate immunity [56]. Grooming is widespread across insects, including social insects 57, 58, and is regarded as a near-universal and evolutionarily conserved strategy of behavioural immunity 59, 60. Allogrooming has been proposed to have been present in the termite ancestor and to represent an early component of termite sociality [57]. Its broad distribution, ancestral origin, and clear role in pathogen removal may therefore help explain why grooming is a prominent focus. That said, substantial biases cause most studies to fail to capture the full repertoires of social immunity mechanisms for any given lineage, making it challenging to infer whether specific traits are ancestral, widespread, derived, or may even have been lost (Figure 2A).

Delving deeper into the focal termite genera that have been explored, our search points to skews that extend well-beyond (sub)families. The genera *Coptotermes* and *Reticulitermes* account for 55% of all studies, and *Nasutitermes*, *Macrotermes*, *Hodotermes*, and *Zootermopsis* are focal in 21–30% of studies (Figure 2B). Skews also extend to pathogen targets, as more than 56% of studies explore responses to *Metarhizium*, *Beauveria*, or *Bacillus* (Figure 2C). Moreover, 88% of studies use microbial strains that do not appear to be native to the studied termite (Figure 2C), such as *Beauveria bassiana*, *Isaria* spp., *Aspergillus* spp., *Candida albicans*, *Staphylococcus aureus*, and *Steinernema*. Most of these have not been verified as natural termite pathogens, but are laboratory models or soil generalists chosen to maximise infection success rather than ecological relevance (see [61]). Only 12% of studies use native genera, such as *Xylaria*, *Fibulorhizoctonia*, and *Athelia,* which are known as symbiotic antagonists or nest moulds (Figure 2C).

When considering social organisation, most studies involve taxa with bifurcated and flexible developmental pathways (56% of studies), followed by those with linear and totipotent strategies (20%) and those with bifurcated pathways, with or without nymphal neotenics, being the least represented (12%) (Figure 2A). This means that current data are insufficient to evaluate predictions across termite taxa that differ in social organisation, not least because only a few studies have integrated social immunity expression and the prospects for direct fitness benefits. To our knowledge, the only exceptions are shifting worker tasks associated with the loss of the soldier caste in *Anoplotermes pacificus* [55] and age polyethism and role shifts in a few other termite species 62, 63, 64, 65, 66. In summary, substantial gaps in our understanding of social immunity in termites and their ecologically relevant pathogens preclude a rigid evaluation of trait connections to social organisation.

## Towards a conditional framework for termite social immunity

### Integrating fitness prospects into a testable framework for the expression of social immunity

The conditional social immunity hypothesis predicts that individuals invest in hygiene when their fitness interests favour doing so [15]. However, termites have a complex organisational system that does not follow strict predictions made for cooperative breeders, and research has rarely included fitness potential when studying social immunity. A central implication of our hypothesis is that termite castes should differ in social immune investment according to their reproductive potential. In lineages with permanently sterile workers, indirect fitness is the only relevant currency, favouring unconditional investment in hygienic care and colony defence. By contrast, in species where workers or nymphs retain the capacity to become neotenic reproductives (e.g., ergatoids), social immunity expression may be flexible because individuals balance collective benefits against future opportunities for direct reproduction [15]. This prediction depends on a more nuanced understanding of reproductive flexibility across taxa, because not all bifurcated or flexible systems produce neotenics, and their frequency may vary among species or even depend on social or ecological contexts.

We predict that social immune investment depends on reproductive prospects, caste-specific constraints, and whether future reproduction is tied to mother-colony survival. (i) Individuals with low or negligible direct fitness potential may show consistently high hygienic responses to pathogen cues, whereas developmentally flexible individuals, including workers, **pseudergates**, or nymphs capable of becoming neotenic reproductives, may express condition-dependent social immunity [15]. (ii) Soldiers may exhibit reduced hygienic behaviours because of morphological constraints, although they may contribute to immunity through defensive or antimicrobial mechanisms [67]. (iii) Nymphs may invest less in collective hygiene than workers, as their future direct fitness prospects are better served by survival and dispersal. Early in their lives, socially dependent nymphs may allogroom, but this behaviour should decline when individuals approach adulthood. Contrasting this prediction, late-stage nymphs have been found to contribute to colony functions, such as tunnel excavation, grooming, brood transport, and participation at foraging sites 68, 69, 70. Hygienic investment by nymphs may thus best be viewed as conditional when it reduces their own infection risk and enhances the survival of colony members.

Comparative analyses that integrate genomic data and behavioural assays can be employed to test whether predicted caste differences in social immune investment scale with deeper evolutionary patterns. Recent genomic work supports this logic, since changes in termite social organisation predicted shifts in gene family evolution and nutrition [33], showing that organisational structure can be used to anticipate how traits are distributed across castes. Although immune gene repertoires appear to have declined over termite evolution 71, 72, particularly within the Termitidae [73], current data is insufficient to determine whether these reductions coincide with the origins of true workers or other organisational transitions. If direct fitness prospects affect social immunity, we expect repeated phylogenetic shifts toward worker-biased expression in lineages where true workers evolved (e.g., superorganisms), and more diffuse or developmentally plastic expression in lineages lacking a permanently sterile worker caste.

### Ontogenetic complexity and potency

Ontogenetic complexity influences how individuals balance the costs of social immune actions. Soldiers, for example, cannot groom but can contribute to social immunity through antimicrobial secretions 67, 74, and they should do so because their fitness returns are exclusively indirect. Workers in bifurcated caste systems add further complexity. If worker-line neotenics are rare, the potential for workers to develop into ergatoid reproductives may be low, such that the cost–benefit ratio of direct versus indirect fitness may be functionally similar to species in which all workers are permanently sterile (‘functional sterility’, *sensu* [18]). Under stable colony conditions, where queen and king dominance suppresses worker-line neotenic development, workers with and without direct fitness prospects are expected to express hygienic care. By contrast, colony instability may alter the reproductive opportunities available to workers. When reproductive dominance weakens, due to ageing or the proliferation of satellite nests, workers with neotenic potential may face opportunities to inherit the colony. These individuals may invest in traits that protect or enhance their own future reproduction, even at the expense of the group. We therefore predict that variation in the likelihood of worker-line neotenic production, rather than developmental potential alone, will influence the conditional expression of costly altruistic sanitary care. Future studies should examine how variation in ontogenetic potency shapes workers’ willingness to perform sanitary care when such strategies are sensitive to future fitness trade-offs.

A feature that may act to stabilise colonies but at the same time favour the production of neotenics is Asexual Queen Succession (AQS), which has been identified in species of the Heterotermitidae 75, 76, 77 and Termitidae 78, 79, 80, 81. In species with AQS, secondary neotenic queens are produced parthenogenetically by the primary queen to increase colony reproductive capacity and longevity 75, 80, 82. The presence of asexual queens may decrease the direct reproductive opportunities available to individual workers, making it important to distinguish worker-line reproductive potential from queen-line succession when examining how ontogenetic pathways shape hygienic expression.

### Colony-age trajectories

In termite clades where workers retain prospects for direct fitness, social immune expression is predicted to change with colony age as the potential for direct fitness changes. The first broods after colony foundation have negligible potential to obtain direct fitness benefits, as this would lead to colony failure, so fitness prospects are exclusively indirect. The queen, king, and the first workers are therefore expected to prioritise investments in social immunity. As the colony grows, investments should adjust accordingly. For example, in *Coptotermes gestroi* [83], brood care is biparental during the first few months and then gradually taken over by workers. As workforce size increases and reproductive opportunities emerge, hygienic investment may increasingly reflect caste-specific fitness interests. Lifelong-sterile workers should continue investing heavily in defences, whereas totipotent workers may reduce group-level contributions if opportunities for direct reproduction increase in older or unstable colonies. These predictions could be evaluated through longitudinal sampling of colonies across developmental stages to test whether hygiene investments shift with colony age.

### Colony mergers

Beyond changes occurring within colonies, interactions between colonies can shape relatedness and individual fitness incentives. Colony fusion has been documented in several termite taxa, although its prevalence remains uncertain, with fusion likely to be conditional 84, 85, 86. Because mergers reduce average relatedness among colony members, indirect fitness benefits of cooperative investment may decline, particularly for individuals that lose their parents in the process. Nevertheless, recent models of soldier investment predict that the consequence of fusion may not be determined by relatedness alone [87]. After fusion, defensive castes and cooperative behaviours may function as public goods: their benefits are shared across the group, whereas costs may be disproportionately paid by the source colony that invested in these traits. Thus, after fusion, investment in soldiers or hygienic defence may create opportunities for exploitation if one colony benefits by reducing its own investment. This may cause mergers to involve reproductive competition, aggression, and suppression of rivals, with selfish strategies dominating even under elevated risks of injury or pathogen transmission.

In other cases, colony mergers may result in stable mixed-family colonies in which cooperation persists despite reduced relatedness, as seen in some Termitidae, such as *Silvestritermes euamignathus* 84, 88. Similarly, *Reticulitermes flavipes* workers from experimentally merged colonies groom former nestmates and non-nestmates equally, while increased genetic diversity does not improve survival after fungal exposure [89]. Social immune expression after mergers may be selected for when environmental harshness makes group living advantageous post-merger, whereas colony instability or declining reproductive dominance may favour more selfish behaviours [15]. Comparative field studies are needed to establish how frequently fusion occurs across termite lineages and whether it is sufficiently common to shape the evolution of social immunity.

### Pathogen pressure

Pathogens are major selective forces shaping host defences over evolutionary time [90]. By default, we predict that responses based on social organisation and direct fitness potential should occur irrespective of the pathogen in question, but the type and extent of expression of a trait should depend on pathogen load, virulence, and environmental factors that affect exposure risks and investment in those actions. Foraging biology, diet, nesting substrate, and microbiota diversity may all shape pathogen exposure and colony-level protection. For instance, extranidal foraging can increase contact with pathogen-rich soils [91], while both diet and evolutionary history affect functions obtained from microbial symbionts 92, 93, 94.

Nesting and foraging ecology may further contribute to variation in termite social immunity by affecting pathogen exposure and opportunities for defence. One-piece nesters live and feed within the same piece of wood [95] and thus remain in close proximity to pathogens present in their nesting substrate, while subterranean foragers may encounter a broad range of pathogens through contact with the soil. In contrast, arboreal species may experience comparatively lower exposure to soil-borne pathogens. Nest architecture itself can also affect the expression of hygiene, as nest structures may confer protection [96]. Mound building and compartmentalised nests may act as physical barriers that limit pathogen entry or spread within the colony. Species relying on nest architecture and strong-point defence tend to invest less in soldiers than species employing coordinated counter-attacks [97], likely because structural barriers reduce the need for numerical defensive investment, whereas large nests depend on coordinated actions that favour a greater number of defenders. Whether such variations in soldier investment also extend to pathogen defence remains to be tested. Because nesting strategies and foraging behaviours occur along a continuum rather than as strictly discrete categories 97, 98, variation within and among species may generate corresponding differences in social immune expression.

Moreover, nest-associated microbiota may buffer pathogen pressures, as nest structures can harbour specialised microbial communities that are distinct from surrounding soils, guts, or other substrates [99]; faecal or carton nest materials and symbiotic bacteria can inhibit antagonistic microbes and contribute to colony hygiene 100, 101, 102. If colony protection is accomplished through other means, social immunity expression may be lower in individuals and hence less dependent on reproductive potential. Future studies should assess the balance between reproductive potential and ecological aspects across termite taxa, including how microbial roles affect conditional social immunity.

Lastly, high pathogen load or high virulence may shift totipotent worker decisions [15], as they could alter the prospects for future direct or indirect fitness benefits. For instance, disease outbreaks could lead totipotent workers to suppress selfish behaviours to invest more in group-level cleaning, or they could abandon the nest and start a new colony if environmental conditions are favourable. Future work should test how infection regimes and disease outbreaks affect decision-making when workers face alternative fitness-maximising scenarios. In addition to selfish workers becoming reproductives [103], pathogen pressure may also influence the likelihood of colony mergers. While we are aware that this may not improve survival against pathogens, as seen in *R. flavipes* [89], it is still not universally clear whether increased pathogen pressures increase the propensity for colonies to merge. Overall, pathogen pressure is expected to interact with social organisation in context-dependent ways, such that reproductive incentives, pathogen traits, foraging ecology, nest structure, and microbial defences jointly determine the expression of a given social immune trait.

## Concluding remarks

Our conceptualisation of social organisation in termites exemplifies the marked differences from those in social Hymenoptera that must be considered to generate robust predictions of costly altruistic acts, including those in social immunity. By harnessing insights from termite social evolution, our framework allows experimental testing in a phylogenetic context, permitting explicit, hypothesis-driven investigation of the expression and evolution of social immune traits, their ecological and evolutionary constraints, and their potential status as evolutionary novelties (see Outstanding questions). Our analysis also illustrates three major priorities for future work: (i) the need to use ecologically relevant pathogens, (ii) the need to consider substantial gaps in social immune research across termite clades, particularly within the Termitidae, and (iii) the general lack of comparative analyses. We hope that this framework provides motivation and increased opportunity for improving comparative analyses to strengthen our understanding of the evolution of social immunity in termites. More broadly, the diversity of extant social organisation across termites allows for a series of natural experiments, making the clade a powerful model for testing general hypotheses about the evolution of altruistic hygienic traits.

Outstanding questions How do caste-specific fitness interests and differences in reproductive potential shape the evolution, expression, and distribution of social immune behaviours across termite lineages with different forms of social organisation (Figure 1)?

To what extent do differences in developmental potential, reproductive opportunities, and caste-specific physiological constraints determine when individuals prioritise collective disease defence over future direct fitness, and how do termite colonies maintain effective social immunity when some individuals have incentives or limitations that reduce their participation in hygienic care?

Is social immune investment in termites developmentally fixed or flexibly adjusted according to physiological costs, environmental conditions, and reproductive potential?

How does colony age alter caste-specific investment in social immunity as direct and indirect fitness opportunities shift over colony development (e.g., in totipotent and bifurcated/flexible systems)?

How do colony mergers alter social immune investment by reshaping relatedness, reproductive opportunities, and the balance between cooperation and selfishness in termite societies?

To what extent do pathogen load, virulence, and infection risk modify fitness-maximising strategies in termites, shifting individuals between selfish reproductive behaviours and cooperative social immune investment, and under what conditions can pathogen pressure reshape colony organisation, conflict, and colony merging?

Alt-text: Outstanding questions

Key references Cremer S. and Pull C.D. (2024) Unconditional versus condition-dependent social immunity. *Trends Parasitol.* 40, 780–787. 10.1016/j.pt.2024.07.014

This work provides the concept for distinguishing between unconditional and conditional social immunity based on the balance between direct and indirect fitness. Our review extends this framework to termites, arguing that developmental plasticity and variation in reproductive potential among nonreproductives generate predictable differences in social immune expression that are not captured by existing broad classifications.

Revely L. *et al*. (2021) The plasticity and developmental potential of termites. *Front. Ecol. Evol.* 9, 552624. 10.3389/fevo.2021.552624

This review synthesises evidence for developmental plasticity and reproductive flexibility across termite lineages. It provides the biological foundation for our argument that variation in ontogenetic potential influences the fitness consequences of altruistic behaviour and that it should be incorporated into predictions of social behaviour (e.g., social immunity).

Hellemans S. *et al.* (2024) Genomic data provide insights into the classification of extant termites. *Nat. Commun.* 15, 6724. 10.1038/s41467-024-51028-y

This study provides the most comprehensive phylogenomic framework for extant termites to date. By resolving relationships among major termite lineages, this study enables us to conduct comparative analyses of social organisation and social immunity, forming a key basis for the evolutionary predictions developed in our framework.

Haifig I. *et al*. (2016) Unrelated secondary reproductives in the neotropical termite Silvestritermes euamignathus (Isoptera: Termitidae). *Sci. Nat.* 103, 9. 10.1007/s00114-015-1325-0

This study demonstrates that reproductive opportunities in termites can extend beyond close kin relationships, highlighting the complexity of fitness interests within colonies. It provides empirical support for considering direct fitness potential when predicting the expression of altruistic traits, including social immunity.

Roisin Y., Korb J. *et al*. (2010) Social organisation and the status of workers in termites. In *Biology of Termites: A Modern Synthesis* (Bignell D.E. *et al.* eds), pp. 133–164, Springer Netherlands

This chapter provides the conceptual basis for distinguishing termite social systems on the basis of worker status, developmental flexibility, and reproductive potential. It is central to our framework because these organisational differences underpin predictions about the conditional versus unconditional expression of social immunity.

Alt-text: Key references

## Author contributions

E.L.C. and M.P. jointly developed the conceptualisation for this review. A.C.G., E.L.C., I.H., and M.P. wrote the original draft of the manuscript. All authors contributed to the critical review and editing of the final version. All authors have read and approved the final manuscript.

## Glossary

**Alate**: a primary reproductive individual with functional eyes, sex organs, and wings that has not yet left the natal nest to participate in a mating flight and found a new colony.
**Ergatoid neotenic**: an individual with reproductive potential that develops from a worker line (–wing buds). These individuals retain juvenile or worker-like characteristics (neoteny) and function as replacement or supplementary reproductives, although this is thought to be rare.
**Hemimetabolous**: insects that develop through incomplete metamorphosis, with individuals being smaller and wingless and gradually acquiring adult features through successive moults without a pupal stage.
**Inclusive fitness**: the combined fitness of an individual derived from its own direct reproductive success via offspring production and from indirect fitness acquired by helping kin reproduce (refer to Box 1).
**Major evolutionary transition**: a key event in the history of life in which previously independent biological units merge to form more complex, higher-level organisms or systems (e.g., the evolution of eukaryotic cells, multicellularity, and superorganismal insect societies).
**Neotenic**: an individual that develops from either the nymphal or worker line (+ or –wing buds) that has functional sexual organs, retains other juvenile characteristics, and does not disperse. A neotenic can either supplement or replace the primary reproductives.
**Nymphoid neotenic**: a secondary reproductive that develops from the nymphal stage and retains juvenile features, such as wing buds, while becoming sexually mature. It can mate and reproduce within the colony and often replaces or supplements the primary reproductives.
**Paedomorphic**: an individual that retains juvenile or larval characteristics into adulthood, even while achieving sexual maturity. In social insects, the term commonly refers to reproductives that develop adult reproductive capacity without fully acquiring typical adult morphology.
**Primary reproductives**: adults (imagos) with functional sex organs, fully developed wings, compound eyes, and sclerotised cuticles that found new colonies and generally mate monogamously; also termed ‘royal pair’ or ‘queen and king’.
**Pseudergate**: a blind, wingless nymph that performs some of the functions of a worker.
**Reproductive altruism**: a form of altruistic behaviour in which an individual reduces or forgoes its own reproduction to assist in the reproductive success of other individuals, typically close relatives, within a social group.
**Subsocial**: social organisation in which parents provide care for their offspring but without forming permanent cooperative groups or castes, or establishing a division of reproductive labour between individuals.
**Social immunity**: collective disease defence in social organisms, whereby group-level traits reduce pathogen transmission and increase the survival of the colony rather than that of individuals alone.
**Superorganismal**: a major evolutionary transition in the evolution of social organisation, involving an irreversible division of labour between sterile and reproductive castes.
**Transgenerational immune priming**: a phenomenon in which parental exposure to an immune challenge results in enhanced immune protection in the offspring, mediated by the transfer of immune factors or epigenetic effects.

## Supplemental information

Text S1 Methodology for the literature review of social immune traits and a list of definitions of social immune traits considered.

Table S1 Overview of the 63 original and review papers that form the basis for the summaries of the three types of social organisation in termites (Figure 1).

Table S2 Overview of original work on social immunity in termites that formed the basis for the summary provided in Figure 2.
