# Supplementary material for: A new narrative for social immunity research in termites
Source: Trends Open. 2026 Oct;1(4):672–85. doi: 10.1016/j.treopn.2026.08.007 (PMC13649642; doi:10.1016/j.treopn.2026.08.007)
Supplement: Supplementary file 1 [file mmc1.docx]

**Supplemental information**

**A new narrative for social immunity research in termites**

Aryel C. Goes^1*^, Erin L. Cole^2*^, Ives Haifig^3^, Dino P. McMahon^4,5^, and Michael Poulsen^2^

*Shared first-authors

^1^Department of Evolution, Ecology and Organismal Biology, Museum of Biological Diversity, The Ohio State University, Columbus, OH 43212, USA

^2^Section for Ecology and Evolution, Department of Biology, University of Copenhagen, 2100 Copenhagen East, Denmark.

^3^Centro de Ciências Naturais e Humanas, Universidade Federal do ABC - UFABC, São Bernardo do Campo, SP 09606-070, Brazil.

^4^Institute of Biology, Freie Universität Berlin, 14195 Berlin, Germany.

^5^Department for Materials and Environment, BAM Federal Institute for Materials Research and Testing, 12205 Berlin, Germany.

Correspondence: mpoulsen@bio.ku.dk (M. Poulsen)

**Text S1**

**Methodology for the literature review in social immune traits**

To investigate whether broad patterns in social immunity traits are consistent with termite social organisation and fitness prospects, we performed a systematic literature review to compile the distribution of 17 reported immune traits across the Isoptera (**given below**). We carried out our search using Isoptera/termite as our main term followed by the individual secondary terms: immunity, social immunity, pathogen, biocontrol, disease defence, and pathogen defence. We selected English-language papers that could be accessed online, through physical libraries, or via personal collections from researchers in the field. Much of the literature on termite immunity is in international journals where English is the primary language used. Where possible, we used reference lists from English- and non-English-language publications to identify additional studies that had been missed in the initial search. If a paper lacked an English abstract, it was excluded. We acknowledge that some relevant literature may nevertheless have been missed, so that our dataset should be interpreted as a structured synthesis of the literature rather than a full global survey. We excluded papers on biocontrol before reaching a final list of 153 studies (**Table S2**). For each study, we identified the trait investigated and noted whether it was confirmed or not. Our evaluation was independent on whether studies addressed social organisation or fitness as predictors for immune expression.

**Definitions of social immune traits across the Isoptera**

Social immune traits identified in our literature review (Table S1). Some definitions reflect study goals more than standardized descriptions; for more detail, see [1–5]. Defensive mechanisms that do not confer protection of the termites themselves, such as the cleaning of fungus gardens in Macrotermitinae, were not considered in this search.

**Aggression:** aggression towards infected individuals, such as leg clipping or killing; at times accompanied by ***cannibalism*** - the consumption of a sick, dying, or dead individual.

**Antimicrobial compound:** bioactive compound from termites or their symbionts, for which antimicrobial activity was evaluated in bioassays or had already been described.

**Antimicrobial materials:** the use of antimicrobial physical structures (e.g., nest material, soil, faeces), with or without bioactive substances or enriched symbiotic microbes.

**Antimicrobial secretions:** bioactive substance(s) from e.g., termite saliva, glandular secretions, or symbionts.

**Avoidance:** detection and recognition of contaminants or sick nestmates leading to reduced social interactions.

**Corpse burial or removal:** undertaking behaviour for corpse management, either as a burial of the corpse using nest materials (e.g., soil boluses, faeces) or physical transport away from the nest.

**Genes involved in defence:** identified and annotated genes with the function being described or predicted to participate in individual immune pathways or in the behavioural/physiological mechanisms of social immunity.

**GNBP (Gram Negative Binding Protein):** pattern-recognition proteins that bind to microbial cell-wall components in individual immune response or, particularly GNBP-2, as a mediator of collective defence behaviours.

**Grooming:** physical removal of e.g., spores from an individual’s body as a prophylactic measure against pathogens. This category also includes allogrooming - nestmate(s) grooming another individual’s body surface.

**Immune gene expression:** gene expression changes associated with infection or individual/social immune responses.

**Pathogen alarm behaviour:** communicative behaviour triggered by cues from pathogens or sick individuals, including, but not limited to, longitudinal oscillatory movements (LOMs).

**Social environment:** group living contributing to better individual survival in the presence of pathogens, without determining which defensive trait is responsible.

**Social immunization:** immune priming from exposure to a sub-lethal pathogen dose, enhancing resistance for the individual or the group in subsequent exposures.

**Social network:** changes in interaction between colony members, their organisation, and/or resource exchanges upon infection that affect social immunity expression.

**Soldiers:** the caste specialized for protection that has been shown to play a role in social immunity, e.g., through the production of volatiles or antimicrobial secretions.

**Symbiont-mediated:** symbiont-derived antimicrobial compounds used in connection with termite immune responses.

**References**

S1 Liu, L. et al. (2019) The Mechanisms of Social Immunity Against Fungal Infections in Eusocial Insects. *Toxins* 11, 244.

S2 Rosengaus, R.B. et al. (2010) Ecology, Behavior and Evolution of Disease Resistance in Termites. In *Biology of Termites: a Modern Synthesis* (Bignell, D. E. et al., eds), pp. 165–191, Springer Netherlands.

S3 Masri, L. and Cremer, S. (2014) Individual and social immunisation in insects. *Trends in Immunology* 35, 471–482.

S4 Stroeymeyt, N. et al*.* (2014) Organisational immunity in social insects. *Current Opinion in Insect Science* 5, 1–15.

S5 Hassan, A. *et al.* (2021) Alteration of Termite Locomotion and Allogrooming in Response to Infection by Pathogenic Fungi. *Journal of Economic Entomology* 114, 1256–1263.
